# The Ontario Electronic Consultation (eConsult) Service: Cross-sectional Analysis of Utilization Data for 2 Models

**DOI:** 10.2196/32101

**Published:** 2022-04-22

**Authors:** Sheena Guglani, Clare Liddy, Amir Afkham, Rhea Mitchell, Erin Keely

**Affiliations:** 1 C.T. Lamont Primary Health Care Research Centre Bruyere Research Institute Ottawa, ON Canada; 2 Department of Family Medicine University of Ottawa Ottawa, ON Canada; 3 eConsult Centre of Excellence The Ottawa Hospital Ottawa, ON Canada; 4 Ontario Health East Ottawa, ON Canada; 5 Department of Medicine University of Ottawa Ottawa, ON Canada; 6 Division of Endocrinology and Metabolism The Ottawa Hospital Ottawa, ON Canada

**Keywords:** eConsult, access to care, utilization, consultation, primary care provider, direct-to-specialist, Ontario, healthcare system

## Abstract

**Background:**

The Ontario electronic consultation (eConsult) service allows a primary care provider (PCP) to access specialist advice through 2 models: the direct-to-specialist (DTS) model, where PCPs select a specialist from a directory, and the Building Access to Specialists Through eConsultation (BASE)–managed specialty service, where PCPs choose a specialty group and are assigned a specialist from a qualified pool based on availability.

**Objective:**

The aim of this study is to examine patterns of use between the 2 models of eConsult delivery.

**Methods:**

We conducted a cross-sectional analysis of utilization data collected from eConsults completed between October 2018 and September 2019. Cases were grouped based on the model used for submission (ie, BASE or DTS). Each model was assessed for the number of cases over time, specialty distribution, proportion resulting in new or additional information, impact on PCPs’ decisions to refer, and billing time.

**Results:**

PCPs submitted 26,121 eConsults during the study period. The monthly case volume increased by 43% over the duration of the study, primarily in the BASE model (66% compared to 6% for DTS). PCPs were able to confirm a course of action that they originally had in mind in 41.4% (6373/15,376) of BASE cases and 41.3% (3363/8136) of DTS cases and received advice for a new or additional course of action in 54.7% (8418/15,376) of BASE cases and 56.3% (4582/8136) of DTS cases. A referral was originally contemplated but avoided in 51.3% (7887/15,376) of BASE cases and 53.3% (4336/8136) of DTS cases, originally contemplated and still needed in 19.4% (2986/15,376) of BASE cases and 17.7% (1438/8136) of DTS cases, and neither originally contemplated nor needed in 21.7% (3334/15,376) of BASE cases and 21.9% (1781/8136) of DTS cases.

**Conclusions:**

Both eConsult models had strong uptake. Use patterns varied between models, with the majority of growth occurring under BASE, but survey responses showed that both models provided similar outcomes in terms of new information offered and impact on decision to refer.

## Introduction

Canadians face prolonged wait times for specialist care in comparison to other developed countries. In Canada, patients do not access specialty care directly in most cases. Rather, they must first see a primary care provider (PCP), a family physician or nurse practitioner, who completes an assessment and refers them to a specialist, often one in their professional network or with whom they have had a positive experience [1-4]. Once the referral is complete, patients must wait for an appointment, which for some specialties can take months or even years [5,6]. However, electronic consultation (eConsult) can greatly improve access to specialist advice in many cases by allowing requesting providers, usually PCPs, to communicate directly with specialists electronically regarding a patient’s care, often avoiding the need for a traditional consultation. Studies of eConsult services have found that they improve access, lower costs, and deliver high rates of patient and provider satisfaction [7,8].

As early as 2010, regional services in Ontario provided access to specialist advice for PCPs operating in their jurisdictions [9]. However, the availability of these services varied geographically, with some PCPs limited in their ability to access services in their regions. This changed in 2018, when Ontario’s Ministry of Health supported the creation of the Ontario eConsult Service, building on existing programs and expanding their reach across the entire province [10].

The Ontario eConsult Service provides access to 2 models of multispecialty provider-to-provider eConsult. In the direct-to-specialist (DTS) model, PCPs select an individual specialist from a directory and send their question to them directly via the eConsult platform [11]. In the Building Access to Specialists Through eConsultation (BASE)–managed specialty service model, PCPs select a specialty group from a menu and the case is then assigned to an individual specialist by the operations team, which has oversight of availability, timeliness, and the specialists added to the pool [9]. To our knowledge, this is the only eConsult service that offers PCPs 2 models of accessing specialist advice on one eConsult platform [12,13].

In this study, we aimed to identify and compare patterns of use between the 2 models of eConsult delivery available through the Ontario eConsult Service. A better understanding of how PCPs use each model will help inform the service’s continued expansion and provide insight for innovators looking to establish an eConsult service tailored to their jurisdiction’s needs.

## Methods

### Design

To identify patterns of use between models, we conducted a cross-sectional analysis of utilization data emerging from the Ontario eConsult Service.

### Setting

The Ontario eConsult Service operates in Canada’s most populous province, with a population of over 14 million people (nearly 40% of all Canadians) [14] served by approximately 15,000 family physicians (ie, those with general practice as their primary or secondary specialty) [15] and 4000 registered nurse practitioners [16]. Like all Canadian provinces, Ontario oversees a federally funded but provincially run Medicare program, which provides health care services to residents free of charge. At the time of this evaluation, the province was divided into 14 health regions known as Local Health Integration Networks (LHINs). Each LHIN coordinated health care services for the needs of its unique population. As of 2020, this model was phased out and the 14 LHINs were reorganized as 5 regions.

### The Ontario eConsult Service

The Ontario eConsult Service was launched in June 2018 and is supported by the province’s Ministry of Health. Prior to the launch of the new service, there were 2 provincial services with different models of specialist access. The Champlain BASE service, which began as a regional pilot project in the Champlain LHIN (comprising Ottawa and Eastern Ontario) in 2010 before expanding to other regions, notably the South East LHIN, provides specialist access through the managed specialty service model. The Ontario Telemedicine Network (OTN) was launched in 2012 in Toronto and uses the DTS model. The newly formed Ontario eConsult Service was launched on the OTN and includes both specialist access models. Both services are provider-to-provider, meaning that patients do not use them directly, but PCPs submit questions to specialists concerning their care. It is the PCP’s choice which way they access a specialist for each case.

All practicing specialists in Ontario can join the service through the DTS model. BASE operates using a pool of specialists recruited based on need, geographic location, and experience. The number of specialists in each specialty group ranges from 2 to 18 (Multimedia Appendix 1). Specialists in a BASE group may also participate in the DTS option.

**Figure 1:**
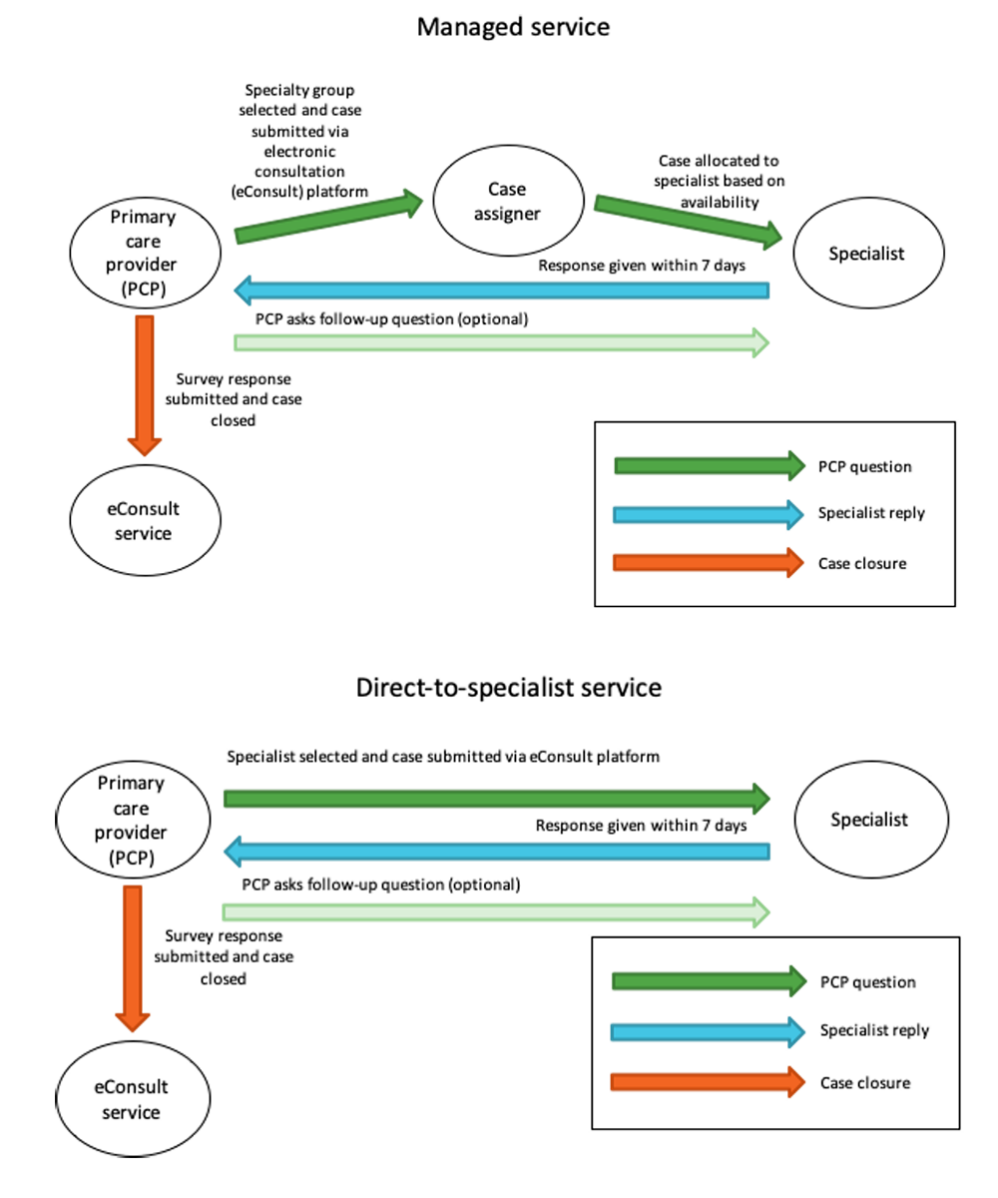
Workflow chart for electronic consultation cases submitted through the Building Access to Specialists Through eConsultation (BASE) and direct-to-specialist models.

The Ontario eConsult Service operates at no charge to PCPs and patients. Specialists are remunerated at an hourly rate prorated to their self-reported billing time. PCPs are also remunerated via the publicly funded Ontario Health Insurance Plan at a flat rate per case. To use the service, PCPs log into the site via an OTN account, select the eConsult model they want to use (DTS or BASE), choose a specialist (DTS) or specialty group (BASE), enter their question, and submit (Figure 1). DTS cases are sent to the specialist; under the BASE model, a case is assigned to a specialist within the chosen group, ensuring cases are evenly distributed among the group’s specialists while taking into consideration current availability, desired case volume, and other special factors (eg, specialists who only see patients of a certain age or from a specific region). To build communities of practice, BASE operates regionally where possible, referring PCP questions to specialists operating in their region.

In either model, the specialist receives a notification and responds to the question within 1 week by providing guidance, recommending a referral, or requesting more information. Discussion can proceed iteratively back and forth until the PCP is satisfied with the response, at which point they complete a mandatory closeout survey assessing the case’s outcome, impact on decision to refer, and educational value. An optional free-text field allows for additional comments.

### Outcomes

Cases were grouped based on the model used for submission (BASE or DTS). Each model was assessed for the number of cases over time, proportion of cases resulting in new or additional information, impact on PCPs’ decisions to refer, and billing time.

### Data Collection

We used routine utilization data automatically collected for each case. This includes user ID, region, billing time, cost, specialty, specialist, and results of the mandatory closeout survey asking PCPs to identify (1) whether the response confirmed their original course of action or provided them with new or additional information and (2) what impact it had on their decision to refer the patient. Our team extracted data from all cases submitted over a 1-year period between October 2018 and September 2019. Subspecialties from both models were grouped into the most relevant specialty (eg, cases sent to pediatric dermatology were grouped as dermatology).

### Data Analysis

We conducted a descriptive analysis (eg, mean, median, count, and distribution) to compare metrics between models of care. A time series analysis was conducted to observe growth and changes in the proportion of cases provided to the DTS model compared to the BASE model. Data were grouped by each PCP’s LHIN to observe trends in different regions of the province. Additional statistical analyses for time billed and response intervals were performed using the statistical software package SPSS Statistics (version 27.0; IBM Corp). Time billed at or under 25 minutes by specialists (in discrete time blocks of 5, 10, 15, 20, and 25 minutes) and time billed over 25 minutes (continuous variable) were analyzed independently. The Kolmogorov-Smirnov and Shapiro-Wilk tests were used to test the normality of the data. Due to the data not being normally distributed, the nonparametric Mann-Whitney U test and Pearson chi-square test were used to assess the differences between groups.

### Ethics Approval

This project was approved as a quality improvement initiative by the Ottawa Hospital Health Sciences Network Research Ethics Board.

## Results

PCPs submitted 26,121 eConsults to the Ontario eConsult Service during the study period, out of which 24,178 eConsults were responded to by a specialist during the study period. (Table 1). A total of 65% (16,985/26,121) of cases were submitted through the BASE model. A total of 2880 requesting providers submitted at least 1 eConsult, of whom 39.3% (n=1133) used BASE exclusively, 19.1% (n=551) used DTS exclusively, and 41.5% (n=1196) used both. Of the 2880 requesting providers, 27.8% (n=801) submitted 10 or more eConsults during the study period. Among this smaller, high-volume user group, 21.2% (170/801) used BASE exclusively, 5.2% (42/801) used DTS exclusively, and 73.5% (589/801) used both models.

**Table 1:** Comparison between BASE^a^ and DTS^b^ models.

Electronic consultation model	Cases submitted (N=26,121), n (%)	Mean time billed in minutes	Response interval in days	Referral avoidance, n (%)^c^	Referral initiation n (%)^c^	Cases cancelled or declined (n (%)^d^
BASE	16,985 (65)	17.5	1.14	7887 (51.3)	494 (3.2)	749 (4.4)
DTS	9136 (35)	21.7	0.99	4336 (53.3)	257 (3.2)	261 (2.9)

^a^BASE: Building Access to Specialists Through eConsultation.

^b^DTS: direct-to-specialist.

^c^For this category, n=15,376 for BASE and n=8136 for DTS; percentages have been calculated accordingly.

^d^For this category, n=16,985 for BASE and n=9136 for DTS; percentages have been calculated accordingly.

**Figure 2:**
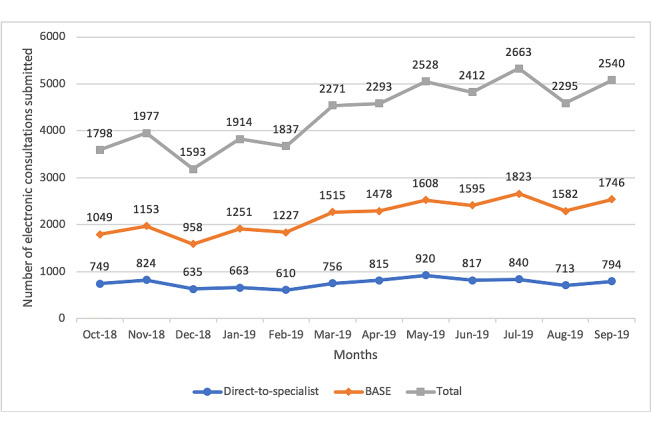
Historical time series of cases submitted to the Building Access to Specialists Through eConsultation (BASE) and direct-to-specialist models.

The number of eConsult cases submitted increased by 41% over the duration of the study (Figure 2). Most of this increase occurred for BASE, which saw a 66% increase in use compared to a 6% increase in the use of DTS. The service also demonstrated a growth in the number of active users, defined as those who submit or answer 3 or more eConsults in a 6-month period. The service saw a 48% increase in active PCPs and a 28% increase in active specialists.

**Figure 3:**
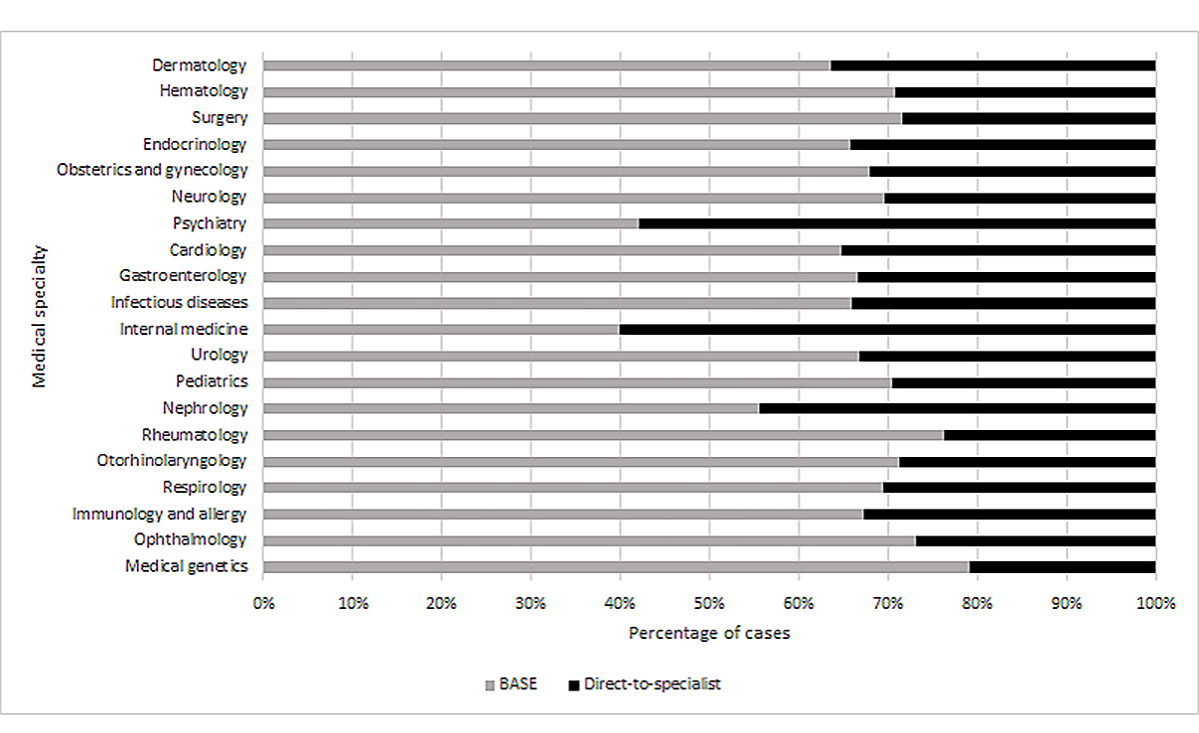
Proportion of cases involving the 20 most frequently accessed specialties for the Building Access to Specialists Through eConsultation (BASE) and direct-to-specialist models.

**Figure 4:**
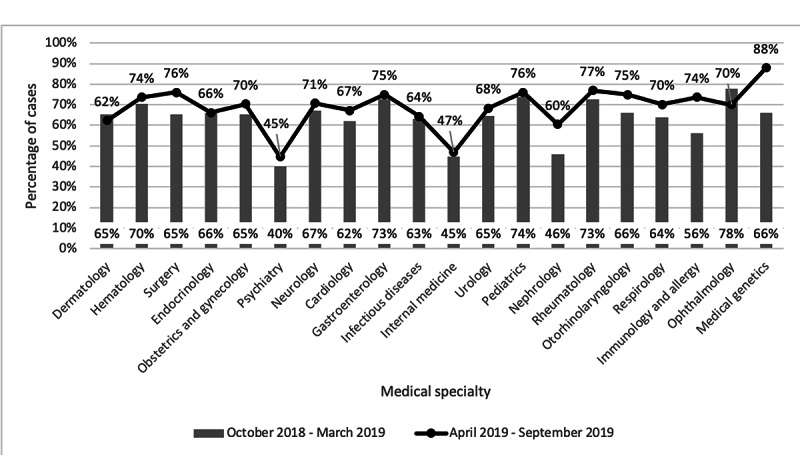
Comparison of the percentage of Building Access to Specialists Through eConsultation (BASE) cases provided for the top 20 specialties in the first 6 months (October 2018 to March 2019) and last 6 months (April 2019 to September 2019) of the study period.

The 20 most frequently accessed specialties are outlined in Figure 3. Only 2 of the specialties in this group, psychiatry and internal medicine, had a higher proportion of DTS cases than BASE cases. Figure 4 outlines the proportion of BASE cases in the first and last 6 months of the study period for the top 20 specialties.

In 12 of the 14 LHINs, requesting clinicians submitted more cases through BASE than DTS. Only 2 LHINs had a greater proportion of DTS cases compared to BASE cases: Erie St. Clair and South West. The Champlain (650/726, 89.5%) and South East LHINs (2575/2972, 86.6%) sent the highest proportion of cases through BASE.

Closeout survey data were available for 23,512 closed cases (Table 1). Based on their responses to the mandatory closeout survey, PCPs had similar experiences across models. PCPs were able to confirm a course of action that they originally had in mind in 41.4% (6373/15,376) of BASE cases and 41.3% (3363/8136) of DTS cases and received advice for a new or additional course of action in 54.7% (8418/15,376) of BASE cases and 56.3% (4582/8136) of DTS cases. When asked about each case’s impact on their decision to refer, PCPs stated that a referral was originally contemplated but avoided in 51.3% (7887/15,376) of BASE cases and 53.3% (4336/8136) of DTS cases, originally contemplated and still needed in 19.4% (2986/15,376) of BASE cases and 17.7% (1438/8136) of DTS cases, and neither originally contemplated nor needed in 21.7% (3334/15,376) of BASE cases and 21.9% (1781/8136) of DTS cases.

Among cases with billing times of 25 minutes and under, specialists responding through DTS had a higher mean billing time (14.12 minutes) than those responding through BASE (13.96 minutes; *χ^2^*_5, N=21,768_=80.471; *P*<.001). Time billed over 25 minutes also differed significantly between models, with cases provided through DTS having a mean billing time of 45 minutes compared to a mean of 37 minutes for BASE (*U*=1,570,925; *Z*=13.326; *P*<.001; *r*=0.24). The response interval of cases provided through DTS (median 0.99 days) was slightly lower than the response interval for cases provided through BASE (median 1.14 days; *U*=63,495,746; *Z*=–9.6; *P*<.001; *r*=–0.06).

## Discussion

### Principal Results

The Ontario eConsult Service processed over 26,000 cases across 2 models of care, registering growth in the number of monthly cases (41% increase), active PCPs (48% increase), and active specialists (28% increase). Two-thirds of cases were provided through the BASE model. PCPs who used the service frequently tended to use both avenues to access specialty advice. Cases provided through the DTS model had a longer median billing time and a shorter median response interval.

### Exploration of Findings

The BASE and DTS models each offer advantages and drawbacks. In the BASE-managed service model, eConsult requires a smaller number of specialists; for some less commonly used specialty groups, 2 specialists are sufficient to handle case volume. As a consequence, the frequency of cases sent to a specialist can be adjusted, ensuring that a given provider does not become overwhelmed by demand but also receives enough cases to keep them in practice with the platform. The BASE model also allows PCPs from underserved areas to gain access to advice from specialists practicing in different parts of the province. This is vital to ensuring equity of access and of particular importance to rural and remote patients, many of whom must travel many hours for in-person specialist appointments. However, a drawback to the model is that PCPs cannot choose which specialist their case will be assigned to, which they may wish to do if they have a past working relationship with a particular specialist or if they know the specialist is familiar with their patient’s condition. In these cases, DTS offers a distinct advantage as PCPs can reach out to a particular specialist in their region when available. This direct connection facilitates collegiality and makes it easier for patients to see a specialist who has already communicated with their PCP about their issue in the event the PCP decides to refer them. For a PCP who does not have a prior relationship with a practitioner from a given specialty, the choice of a particular specialist may actually be more difficult than relying on a central case assigner. Fortunately, the various advantages and drawbacks of each model make them complementary, allowing PCPs to choose the service that best fits their situation. It was this in large part that caused our team to incorporate both models as this allowed users to select the service type that best met their needs.

The proportion of cases sent using BASE compared to DTS varied between specialty groups and regions. In some cases, the discrepancy in use patterns between regions may be structural. For instance, the 2 LHINs with the highest proportion of BASE cases, South East and Champlain, have regional BASE groups that predate the Ontario eConsult Service; the South East LHIN launched eConsult in 2015, while BASE first began as a pilot program in Champlain in 2010 and continues to operate in the region outside of the Ontario eConsult Service. As such, it is perhaps not surprising that PCPs who had already used BASE for years would continue to use that model under the Ontario eConsult Service. However, the broader use pattern goes beyond this explanation and may be affected by how many specialists from a given group practice in the PCP’s region. Although all but the most remote communities in Ontario have practicing PCPs, specialists tend to practice in higher-density urban areas, where a larger population size can better support a narrower scope of practice. As a result, PCPs in regions with fewer local specialists may be more likely to use the BASE model as it allows them to access advice through the provincial service in cases where local specialists are unavailable. Additionally, it is worth noting that of the 20 most frequently used specialties, only 2 were accessed predominantly through DTS: psychiatry and internal medicine. This may speak to the importance of established relationships and communities of practice in these specialty groups.

Most of the growth on the Ontario eConsult Service was a result of an increase in the use of the BASE model, which accounted for two-thirds of all cases. We hypothesize the following reasons for the growth of the BASE model: (1) This model eliminates dependency on a PCP having an established network of individual specialists by providing a network of specialty services to access. This promotes equity of access and is especially advantageous for new graduates or a physician who has relocated to a new area. (2) The low resourcing required to launch a BASE group (ie, only 2 specialists are needed to launch a group) allows for a large offering of specialty and subspecialty groups that meet PCPs’ wide variety of needs. Finally, (3) BASE has an easy workflow and intuitive user interface, making it simple to adopt and use. Our data indicate that the growth of the BASE models was not accounted for by a specific specialty (Figure 4) or by the cancellation rates (Table 1). Further study to understand the influencing factors on BASE model use, such as a PCP’s year of graduation or impacts of promotional activities by the eConsult team on DTS versus BASE use, should be explored.

### Limitations

Our study has several limitations. Though focusing on Canada’s most populous province across a wide geographical area, our data set nevertheless can account for only one segment of the country, and results therefore may not be generalizable nationally or in other countries. The study relied on utilization data and survey results. Although useful, these data can provide only an incomplete picture of the service’s use. Other data, including case logs, patient-level data, or electronic medical record surveys would provide more information and should be sought in future studies.

### Conclusions

The Ontario eConsult Service successfully offers 2 models on a single platform. Both models received strong uptake and the service demonstrated growth in cases and levels of adoption by PCPs and specialists during the study period. Use patterns varied between models, with the majority of growth occurring under BASE, but survey responses showed that both models provided similar outcomes in terms of new information offered and impact on decision to refer. DTS and BASE provide complementary benefits, allowing more flexibility for PCPs. Services that are capable of adopting both models should consider offering this option to maximize use and ensure equity of access to prompt and high-quality specialist care.

## Appendix

Multimedia Appendix 1 List of Building Access to Specialists Through eConsultation (BASE) specialty groups, when they were added on the service, and the number of specialists answering electronic consultations (eConsults) in each group.

## Abbreviations

BASE: Building Access to Specialists Through eConsultation
DTS: direct-to-specialist
eConsult: electronic consultation
LHIN: Local Health Integration Networks
MOHLTC: Ministry of Health and Long-Term Care
OTN: Ontario Telemedicine Network
PCP: primary care provider
