# Supplementary material for: The Ontario Electronic Consultation (eConsult) Service: Cross-sectional Analysis of Utilization Data for 2 Models
Source: JMIR Form Res. 2022 Apr 22;6(4):e32101. doi: 10.2196/32101 (PMC9077515; doi:10.2196/32101)
Supplement: Multimedia Appendix 1 [file formative_v6i4e32101_app1.pdf]

BASE™ Specialty Group	Month Year Added	Number of specialists
Provincial Pediatric Dermatology Group	July 2019	4
Provincial Diabetes Group	July 2019	8
Provincial Pediatric Dentistry Group	June 2019	2
Provincial Geriatrics - Frailty Management Group	March 2019	1
Provincial Public Health Group	March 2019	1
Provincial Pediatric Oncology Group	February 2019	1
Provincial Radiology Group	January 2019	3
Groupe provincial de psychiatrie - en francais	December 2018	1
Provincial Cardiology Electrophysiology Group	December 2018	5
Provincial Developmental/Behavioural Psychiatry Group	December 2018	1
Provincial Environmental Health Group	December 2018	2
Provincial Inherited Heart Rhythm Disorders Group	December 2018	3
Provincial Medical Genetics Group	December 2018	4
Provincial Palliative Care Group	December 2018	3
Provincial Pediatric Cardiology Electrophysiology Group	December 2018	2
Provincial Pediatric Hematology Group	December 2018	3
Provincial Pediatric Inherited Heart Rhythm Disorders Group	December 2018	2
Provincial Pediatric Medical Genetics Group	December 2018	2
Provincial Geriatric Medication Group	November 2018	1
Provincial Neurology Headache Group	November 2018	1
Provincial Care of the Elderly Group	September 2018	1
Provincial Developmental Pediatrics Group	September 2018	3
Provincial Neuroradiology Group	September 2018	1
Provincial Pediatric Allergy and Clinical Immunology Group	September 2018	3
**Provincial Pediatric Hepatology Group**	**September 2018**	**2**
Provincial Radiology Breast Imaging Group	September 2018	1
Provincial Thrombosis Group	September 2018	4
Provincial HIV Psychiatry Group	August 2018	1

BASE™ Specialty Group	Month Year Added	Number of specialists
Provincial Medically Complex Psychiatry Group	August 2018	3
Provincial Inflammatory Arthritis Group	August 2018	2
Provincial Male infertility/sexual medicine Group	August 2018	1
Provincial Neuromuscular Group	August 2018	2
Provincial Osteoporosis Group	August 2018	2
Provincial Stroke Group	July 2018	2
Provincial Addiction Medicine Group	June 2018	5
Provincial Allergy & Clinical Immunology Group	June 2018	4
Provincial Anesthesiology Group	June 2018	2
Provincial Breast Oncology Group	June 2018	0
Provincial Cardiac Surgery Group	June 2018	2
Provincial Cardiology Group	June 2018	9
Provincial Concussion Group	June 2018	2
Provincial Dermatology Group	June 2018	9
Provincial Endocrinology Group	June 2018	11
Provincial ENT Group	June 2018	3
Provincial Epilepsy Neurology Group	June 2018	2
Provincial Gastroenterology Group	June 2018	5
Provincial General Pediatrics Group	June 2018	6
Provincial General Surgery Group	June 2018	4
Provincial Geriatric Psychiatry Group	June 2018	3
Provincial Geriatrics Group	June 2018	6
Provincial Gynecologic Oncology Group	June 2018	4
Provincial Gynecology Group	June 2018	7
Provincial Head & Neck Surgery Group	June 2018	3
Provincial Hematology Group	June 2018	7
Provincial Hepatology Group	June 2018	4
Provincial HIV Group	June 2018	5
Provincial Infectious Disease Group	June 2018	11

BASE™ Specialty Group	Month Year Added	Number of specialists
Provincial Internal Medicine Group	June 2018	14
Provincial Medical Oncology Group	June 2018	3
Provincial Neonatal/Perinatal Pediatrics Group	June 2018	1
Provincial Nephrology Group	June 2018	11
Provincial Neurology Group	June 2018	5
Provincial Neurosurgery Group	June 2018	3
Provincial Obstetrics Group	June 2018	6
Provincial Ophthalmology Group	June 2018	2
Provincial Opioid Group	June 2018	3
Provincial Orthopaedic Surgery Group	June 2018	6
Provincial Pain Medicine Group	June 2018	7
Provincial Pediatric Cardiology Group	June 2018	2
Provincial Pediatric Endocrinology Group	June 2018	1
Provincial Pediatric Gastroenterology Group	June 2018	3
Provincial Geriatric Psychiatry Group	June 2018	3
Provincial Geriatrics Group	June 2018	6
Provincial Gynecologic Oncology Group	June 2018	4
Provincial Gynecology Group	June 2018	7
Provincial Head & Neck Surgery Group	June 2018	3
Provincial Hematology Group	June 2018	7
Provincial Hepatology Group	June 2018	4
Provincial HIV Group	June 2018	5
Provincial Infectious Disease Group	June 2018	11
Provincial Internal Medicine Group	June 2018	14
Provincial Medical Oncology Group	June 2018	3
Provincial Neonatal/Perinatal Pediatrics Group	June 2018	1
Provincial Nephrology Group	June 2018	11
Provincial Neurology Group	June 2018	5
Provincial Neurosurgery Group	June 2018	3

BASE™ Specialty Group	Month Year Added	Number of specialists
Provincial Obstetrics Group	June 2018	6
Provincial Ophthalmology Group	June 2018	2
Provincial Opioid Group	June 2018	3
Provincial Orthopaedic Surgery Group	June 2018	6
Provincial Pain Medicine Group	June 2018	7
Provincial Pediatric Cardiology Group	June 2018	2
Provincial Pediatric Endocrinology Group	June 2018	1
Provincial Pediatric Gastroenterology Group	June 2018	3
Provincial Pediatric Infectious Diseases Group	June 2018	3
Provincial Pediatric Nephrology Group	June 2018	3
Provincial Pediatric Neurology Group	June 2018	3
Provincial Pediatric Orthopaedic Surgery Group	June 2018	4
Provincial Pediatric Psychiatry Group	June 2018	3
Provincial Pediatric Rheumatology Group	June 2018	0
Provincial Pediatric Urology Group	June 2018	3
Provincial Perinatal Psychiatry Group	June 2018	2
Provincial Phys Med/Rehab Group	June 2018	4
Provincial Plastic Surgery Group	June 2018	3
Provincial Psychiatric Sleep Medicine Group	June 2018	2
Provincial Psychiatry Group	June 2018	18
Provincial Radiation Oncology Group	June 2018	3
Provincial Respirology Group	June 2018	8
Provincial Respirology Sleep Medicine Group	June 2018	1
Provincial Rheumatology Group	June 2018	8
Provincial Spinal Surgery Group	June 2018	1
Provincial Thoracic Surgery Group	June 2018	2
Provincial Transgender Group	June 2018	4
Provincial Urogynecology Group	June 2018	1
Provincial Urology Group	June 2018	7

BASE™ Specialty Group	Month Year Added	Number of specialists
Provincial Vascular Surgery Group	June 2018	3
Provincial Pediatric Infectious Diseases Group	June 2018	3
Provincial Pediatric Nephrology Group	June 2018	3
Provincial Pediatric Neurology Group	June 2018	3
Provincial Pediatric Orthopaedic Surgery Group	June 2018	4
Provincial Pediatric Psychiatry Group	June 2018	3
Provincial Pediatric Rheumatology Group	June 2018	0
Provincial Pediatric Urology Group	June 2018	3
Provincial Perinatal Psychiatry Group	June 2018	2
Provincial Phys Med/Rehab Group	June 2018	4
Provincial Plastic Surgery Group	June 2018	3
Provincial Psychiatric Sleep Medicine Group	June 2018	2
Provincial Psychiatry Group	June 2018	18
Provincial Radiation Oncology Group	June 2018	3
Provincial Respirology Group	June 2018	8
Provincial Respirology Sleep Medicine Group	June 2018	1
Provincial Rheumatology Group	June 2018	8
Provincial Spinal Surgery Group	June 2018	1
Provincial Thoracic Surgery Group	June 2018	2
Provincial Transgender Group	June 2018	4
Provincial Urogynecology Group	June 2018	1
Provincial Urology Group	June 2018	7
Provincial Vascular Surgery Group	June 2018	3
